# Ultrasound measurements of lumbar spinous process movement during flexion distraction manipulation: a preliminary descriptive cross-sectional study with healthy participants

**DOI:** 10.1186/s12998-025-00593-0

**Published:** 2025-07-25

**Authors:** Ralph Kruse, Maruti Gudavalli, Bret White, Stacey Rider, Dean Greenwood, Casey Rogers

**Affiliations:** 1 Keiser University College of Chiropractic Medicine, West Palm Beach, FL USA; 2 Miami VA Medical Center, Miami, FL USA; 3 Southeastern College, West Palm Beach, FL USA; 4 Greenwood Chiropractic, Vancouver, BC Canada; 5 Birmingham VA Medical Center, Birmingham, AL USA

**Keywords:** Spinal manipulation, Diagnostic ultrasound, Low back pain, Biomechanics, Flexion distraction

## Abstract

**Background:**

Chronic low back pain is a prevalent condition that impairs productivity and quality of life. While spinal manipulative therapy reduces pain and disability, the biomechanical mechanisms underlying these effects remain unclear. This study utilized diagnostic ultrasound to measure lumbo-sacral spinous process movement (L3-S1) during Cox^®^ Flexion Distraction manipulation, Protocol I, providing insight into spinal intersegmental motion.

**Methods:**

This study analyzed a convenience sample of generally healthy participants, aged 21 years and older, from both sexes and various ethnicities who reported no back pain. Participants were recruited through announcements and flyers posted around the Keiser university campus. Data was collected from June-August 2022. The participants were positioned prone on a specialized flexion distraction chiropractic table. Ultrasound imaging was performed to measure the spinous process distance from L3-S1 before the procedure, during flexion distraction utilizing standard Protocol I, and post-procedure. Ultrasound measurements were recorded by identifying the tips of the spinous processes and distances between L3-L4, L4-L5, and L5-S1, before, during, and after flexion distraction. Statistical analyses included paired t-tests to evaluate spinous process distances pre- and during Cox^®^ Flexion distraction, independent t-tests for gender differences, and linear regression for body mass index (BMI) and age correlations with changes in separation distance.

**Results:**

Thirty participants (16 male, 14 female) with a mean age of 32.5 years (Standard deviation [SD] 10.4), mean weight of 69.2 Kg (SD 11.8), mean height of 169.0 cm (SD 8.9), and BMI of 23.9 underwent Cox^®^ Flexion Distraction Protocol I. Spinous process separation increased during treatment: L3-L4 (0.13 mm), L4-L5 (0.13 mm), and L5-S1 (0.16 mm). Paired ttests showed significant pre- and during-treatment changes (*p* < 0.001), with moderate correlations to BMI (R²=0.61) and age (R²=0.58). Gender differences did not reveal statistical differences in separation distances at all lumbar levels measured (*p* > 0.1).

**Conclusion:**

Ultrasound imaging revealed significant separation of spinous processes at L3-L4, L4-L5, and L5-S1 during Cox^®^ Flexion Distraction Protocol I. Statistical analyses showed separation correlated moderately with age and BMI that was unaffected by gender. Future studies should assess this technique’s relevance in patients with low back pain.

## Introduction

Spinal pain is an ubiquitous condition that affects various groups of the population and is among the major reasons for seeking chiropractic or other specialty consultations [1]. Clinicians have access to many imaging modalities for evaluating low back pain (LBP). The application of these modalities primarily depends on the working diagnosis, the urgency of the clinical problem, the availability of the imaging techniques, and the patient’s comorbidities [2–7].

Ultrasound is a versatile and non-ionizing imaging modality used across various medical specialties, including examining spine movement during distraction mobilization and manipulation. Unlike radiography and computed tomography, it does not expose patients to ionizing radiation [8]. Ultrasound is also widely available and cost-effective. Magnetic resonance imaging (MRI) reveals both bony and soft tissue structures in the axial skeleton; however, its use is hampered by the duration of the examination, relatively high costs, limited availability (mainly in tertiary centers), and the contraindications for this diagnostic modality [9].

Ultrasound is a safe, fast, inexpensive, and widely available imaging modality that is well tolerated by patients [10, 11]. Ultrasound enables multiplanar and dynamic examinations of the musculoskeletal system, providing detailed anatomical views of the soft tissues [8]. Ultrasound imaging has been shown to be reliable for the measurement of the distance between lumbar spinous processes [11–14]. Its use is increasing among physicians, and its range of applications in musculoskeletal issues continues to grow [8, 10]. Ultrasound has the potential to enhance the understanding of biomechanical changes observed in cases of lumbar disc herniation [15–18] and ischemic compression [19] by documenting spinal biomechanical changes related to treatment procedures. Therefore, investigating the potential diagnostic use of ultrasound for spinal pain is relevant for clinical practice.

In spinal manipulation and mobilization (SMM) the clinical outcome is influenced by the level of force that is applied. For example, Gudavalli et al. [20], demonstrated the relationship between applied forces and clinical outcomes in patients with cervical pain. In addition, an animal study suggests that low tension traction force might be beneficial for the regeneration and repair of degenerated intervertebral discs [21]. Moreover, another study used magnetic resonance spectrographs to quantify the structural integrity of a degenerated lumbar disc before and after 16 sessions of Cox^®^ Flexion Distraction and found an increase in glycosaminoglycan biosynthesis [22]. Schiopu et al. showed that by restoring the disc height and reducing pressure, glycosaminoglycan levels can be increased in the discs [23]. Furthermore, a preliminary randomized trial reported that low force lumbar distraction provides superior long-term relief for acute lumbar sciatic pain compared to high force [24]. Low force distraction on the lumbar spine relieves pain, while higher force reduces disability [25]. Chiropractors typically evaluate and manage patients with spinal pain using SMM, which includes a specific form known as the flexion distraction technique (FDT). An advanced, evidence-based approach to FDT is Cox^®^ Flexion Distraction (CFD). Of the two protocols for CFD (Protocols I and II), Protocol I, which is used for patients with radiculopathy, was used in this study [26].

Therapeutic benefits associated with FDT include reducing pain and disability in patients with lumbar spinal stenosis [27–32], lumbar intervertebral disc herniation/protrusion, vertebral facet joint syndrome, spondylolisthesis, and scoliosis [3, 26, 33, 34]. It is also effective in increasing intervertebral disc heights in patients with chronic LBP [28, 32–36], and reduces intradiscal pressure in the lumbar spine, likely centralizing the bulged or protruded disc [26–28, 35–37].

The validity of CFD has been explored and described in biomechanical and biochemical studies documenting intradiscal pressure decreases, changes in spinal reflex excitability, alterations in vertebral motion producing increases in disc space height, area, width and spinal mobility, and improvements in intervertebral disc spectral features measured with magnetic spectroscopy to include decreased biochemical pain markers and increased glycoprotein biosynthesis [9, 22, 26, 28]. Recent studies include documentation of the forces delivered during CFD and reduction in the use of opioids in failed back surgical syndrome patients undergoing CFD [22, 28, 32, 37–39]. The reliability of CFD has been presented in multiple case reports, case series, and a randomized clinical trial regarding such spinal conditions as disc herniation, spondylolisthesis, failed back surgical syndrome, spinal stenosis, chronic LBP, radiculopathy [26, 33, 34, 39–46].

The aim of this study was to investigate and measure the movement of the spinous processes in the lumbo-sacral spine (L3-S1) during Cox^®^ Flexion Distraction treatment. To achieve this aim, we used ultrasound to observe and measure the separation of the spinous processes during Cox^®^ Flexion Distraction Protocol I on asymptomatic participants of all sexes and ethnicities, aged 21 years or older.

## Methods

### Study design, and setting

This descriptive cross-sectional study was conducted between June 2022 and August 2022, at Keiser University College of Chiropractic Medicine, West Palm Beach, FL.

### Recruitment and participants

A convenience sample of generally healthy volunteers of all sexes and ethnicities, aged 21 years or older, who denied having LBP participated in this study. The participants were recruited through announcements and flyers that were posted on the Keiser university campus from May 2022 to June 2022. Exclusion criteria included pregnancy, the inability to lie prone for the duration of the procedure, and any discomfort experienced during the procedure. All participants provided written informed consent after discussing their participation in the study. This ultrasound study received ethics approval from the Keiser University Institutional Review Board (IRB000JU21GM99).

### Data collection

**Fig. 1:**
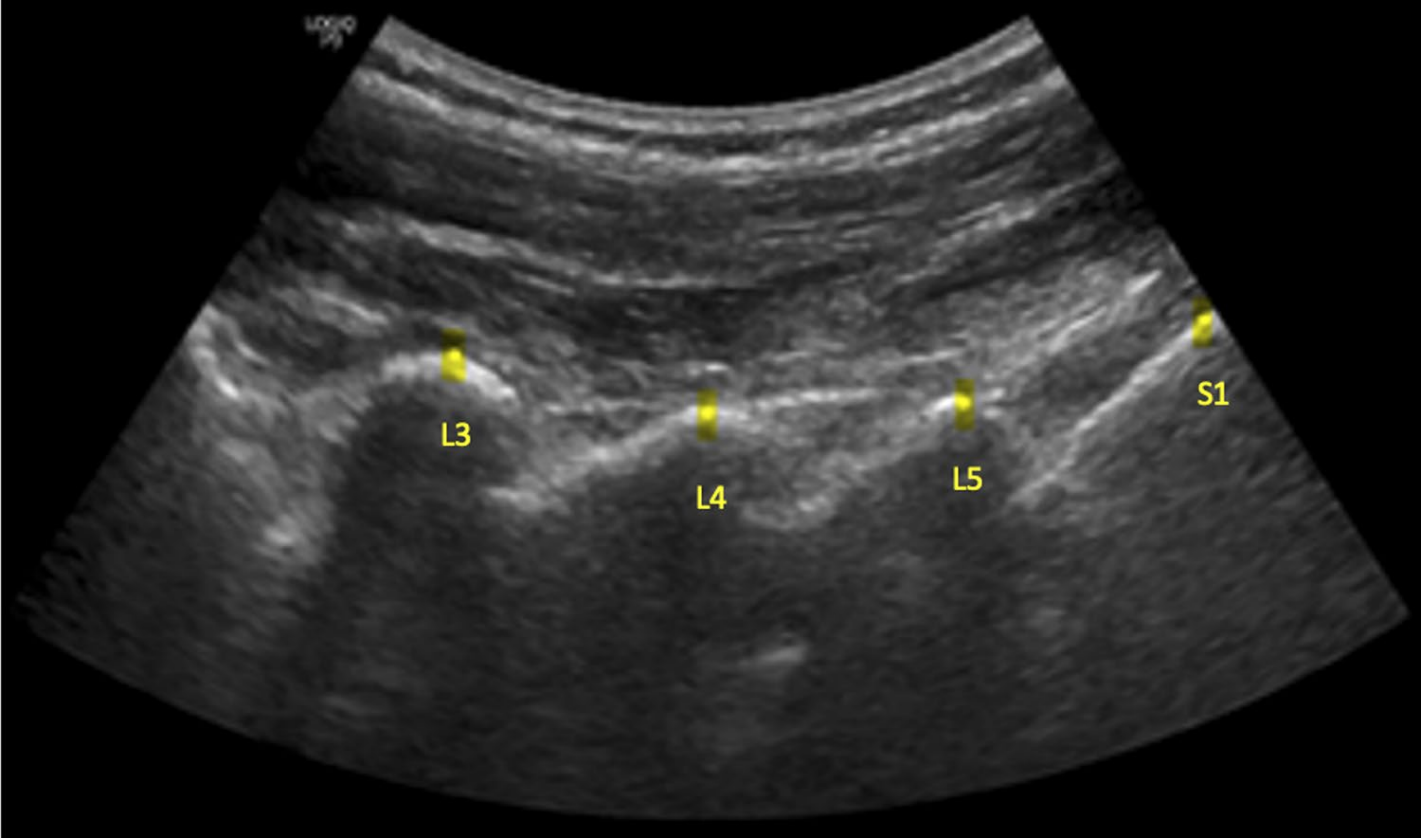
Ultrasound image annotation of the lumbar and sacral regions. Example of ultrasound image annotation of the lumbar region at the lumbo-sacral spine (L3, L4, L5, S1)

**Fig. 2:**
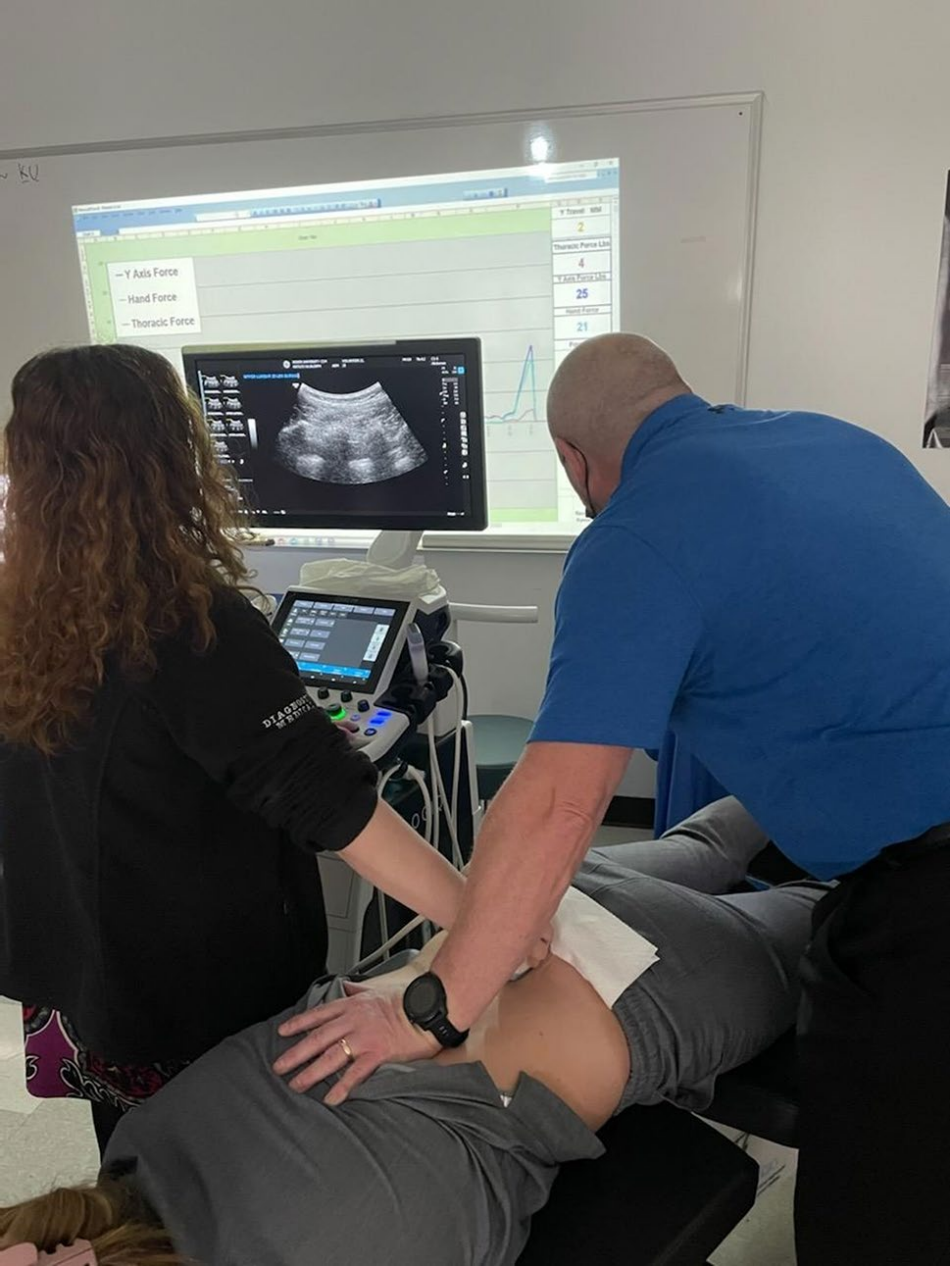
Ultrasound imaging of the lumbar region at the lumbo-sacral spine. The participant is placed prone on the table and is undergoing ultrasound imaging. The chiropractor controls the table’s flexion movements during the procedure

Participants were positioned prone on a flexion distraction table, then ultrasound images and measurements were taken before, during, and one minute after the participants received one session of CFD Protocol I. This protocol was selected because it is the most commonly used protocol in Cox Flexion Distraction. The ultrasound probe was positioned to visualize the lumbar segments, with the clinician’s hand placed just cephalad to the probe. At the point of maximal flexion, the clinician maintained that position while the ultrasound image was obtained (Figs. 1 and 2). The degree of flexion varied among individuals, as the flexion protocol starts when the doctor feels the taut point at the desired spinal segment.

### CFD procedure

The procedure was performed by two licensed chiropractors certified in CFD. The two practitioners carried out CFD as follows. The practitioner placed their hand in a cephalad position superior to the spinous process of interest while implementing a series of five flexion motions, each lasting a total of 4 s (2 s moving inferiorly followed by 2 s returning superiorly) [19]. CFD is the only FDT method with clear treatment protocols, detailing repetitions and sets based on symptoms [26]. Moreover, these protocols improve consistency and validity. Protocol I was used in this study [26]. An automated table with a set degrees of flexion was not used, therefore, the degrees of flexion varied between individuals.

### Ultrasound measurements

Ultrasound (GE LOGIQ P9, GE Healthcare, Chicago, Illinois) was used to obtain images of the spinous processes at L3-L4, L4-L5, and L5-S1 using the curvilinear C 1–5 transducer. Measurements were recorded with the software installed in the ultrasound unit. The distances between the most dorsal aspects of the spinous processes at L3-L4, L4-L5, and L5-S1 were recorded. One ultrasonographer obtained and recorded one measurement per segment for each participant (Fig. 1). The reliability of measuring spinous process distance has been established in previous studies [8, 10].

### Statistics

Demographic data were reported as descriptive statistics (mean, standard deviation [SD]). We used Shapiro-Wilk test and Graphical histograms, and Q-Q plots were used to assess the data for normality. To determine significance, statistical paired t-tests were used to compare the spinous process distances before the manipulation loading and during application of the Cox technique. An independent t-test was conducted to identify any statistically significant differences between male and female participants. Additionally, linear regression analyses were carried out to examine the relationships between body mass index (BMI) and the change in separation distance, as well as between age and the change in separation distance. All statistical analyses were conducted using SPSS v29, IBM Corporation, Chicago, IL.

## Results

### Participants

**Fig. 3:**
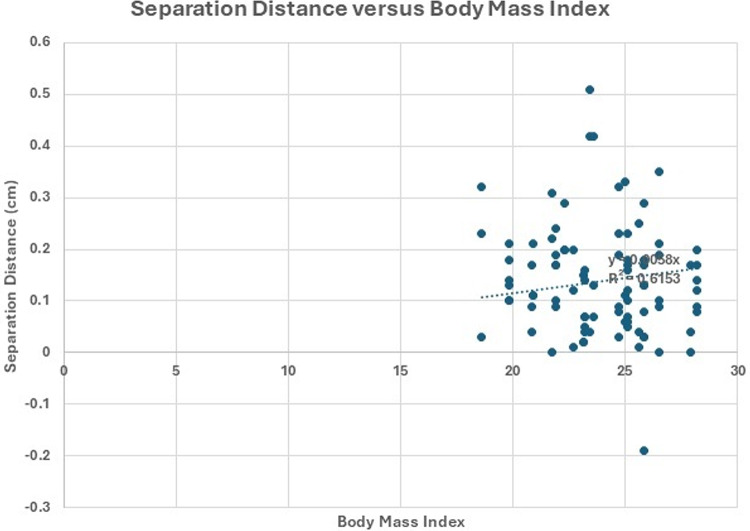
Comparison of body mass index and separation distance for all data (L3-L4,L4-L5,L5-S1) The separation distance versus body mass index is shown along with the linear regression line. The R^2^ value of 0.61 indicates a fair to moderate correlation (*N* = 90)

**Fig. 4:**
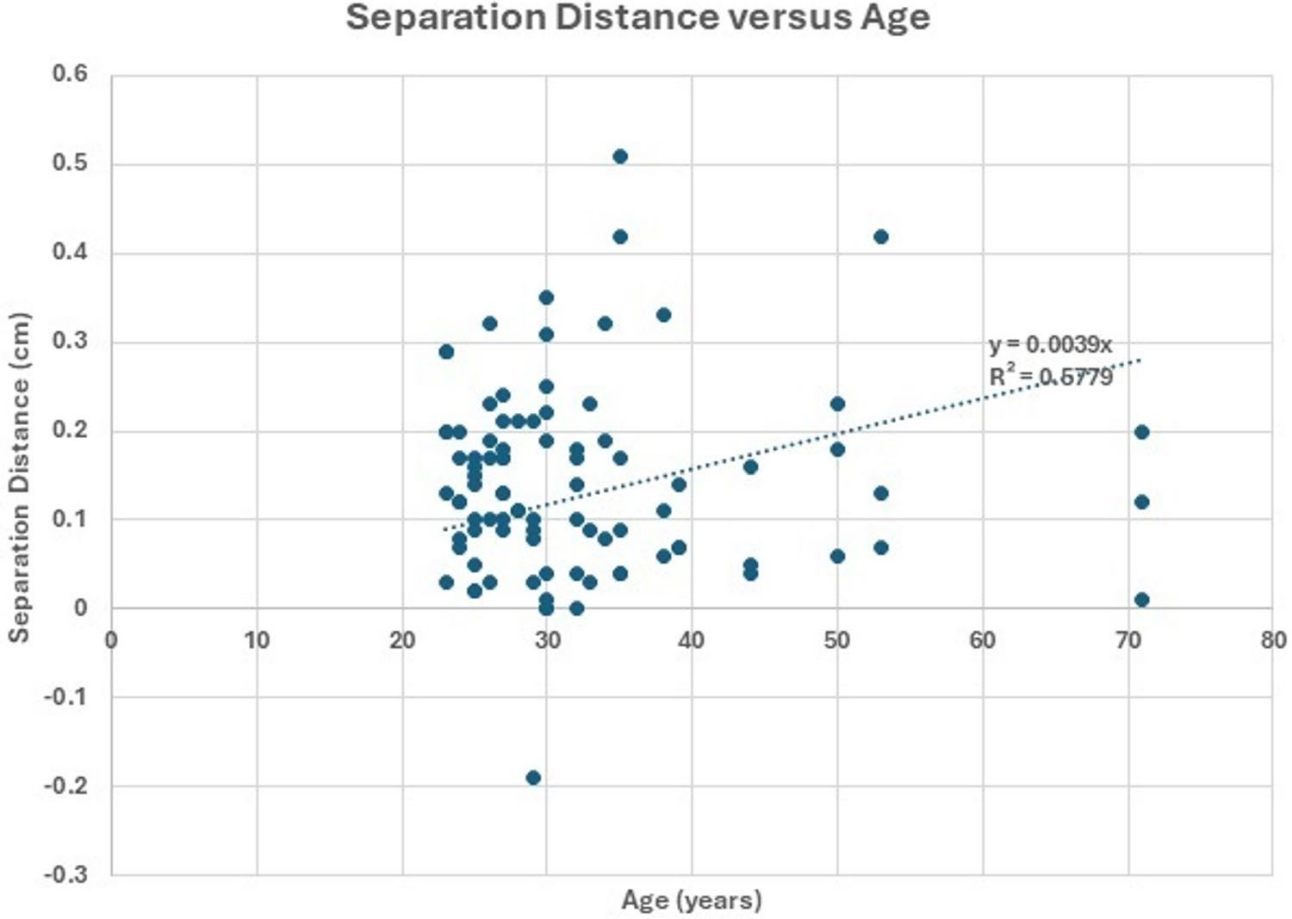
Comparison of separation distance for L3-L4 and age for all data (L3-L4,L4-L5,L5-S1) Age versus body mass index is shown along with the linear regression line. The R^2^ value of 0.58 indicates a fair to moderate correlation (*N* = 90)

A total of 30 participants (16 male, 14 female) underwent CTFDD Protocol I along with ultrasound imaging before, during, and after the procedure. Participant characteristics were as follows: mean age 32.5 years (SD 10.4), mean weight 69.2 kg (SD 11.8), mean height 169.0 cm (SD 8.9), and BMI 23.9. Figures 3 and 4 illustrate the separation distance versus BMI and age, respectively. Figure 3 reports an R² value of 0.61, which signifies a fair to moderate degree of association, suggesting that fluctuations in BMI moderately influence the separation distance. Similarly, Fig. 4 explored the correlation between separation distance and age, and revealed an R² value of 0.58, indicating a similar level of fair to moderate association.

### Spinous process separation during CFD protocol 1

**Table 1:** Spinous processes separation during CTFDD protocol 1

	L3-L4 (mm)		L4-L5 (mm)		L5-S1 (mm)	
	Mean	SD	Mean	SD	Mean	SD
Pre-manipulation	26.59	2.06	24.58	2.99	16.26	2.60
During manipulation	27.85	1.88	25.92	3.02	17.88	2.81
During manipulation to Pre-Difference	1.26	1.06	1.34	1.30	1.62	0.81
Post-manipulation	26.73	1.91	24.57	2.96	16.30	2.60

*N* = 30 participants. Pre-manipulation: Ultrasound measurements were taken before the participant underwent the procedure; During manipulation to Pre-Difference: Ultrasound measurements were taken during the procedure; Post-manipulation: Ultrasound measurements were taken one minute after the procedure.

Table 1 presents the descriptive statistics for the distance between the tops of the spinous processes during CFD Protocol 1. The separation distance between L3-L4 is slightly greater than that between L4-L5 and greater than that between L5-S1. Table 2 displays the descriptive statistics for the spinous process separation at three stages: before, during, and after the participants underwent CFD Protocol 1. During CFD manipulation, the increase in the separation distance is more pronounced at L5-S1 followed by L4-L5, and then L3-L4. After manipulation, the spinous processes returned to their pre-manipulative positions.

Table 2. provides the difference in separation distance between during treatment and the pre-treatment and was found to be statistically significant for all levels as well as individual levels at L3-L4, L4-L5, L5-S1. During treatment separation distance is significantly higher than the pre-treatment value.

**Table 2:** Separation Distance in cm and the t-statistic based on 30 participants

Segment	Pre-Treatment Mean (SD)	During Treatment Mean (SD)	df	t	p
L3_L4	2.66 (0.21)	2.79 (0.19)	29	6.55	<0.001
L4-L5	2.46 (0.30)	2.59 (0.30)	29	5.64	<0.001
L5-S1	1.63 (0.26)	1.79 (0.28)	29	10.92	<0.001
All Segments	2.25 (0.52)	2.39 (0.51)	89	12.43	<0.001

Table 3 provides the difference in separation distance between males and females and was found to be not statistically significant at each of the levels L3-L4, L4-L5, L5-S1.

**Table 3:** Difference in separation distance in cm and the t-statistic based on 30 participants

Segment	Male Mean (SD)	Female Mean (SD)	df	t	p
L3_L4	0.127 (0.091)	0.125 (0.124)	28	0.064	0.95
L4-L5	0.098 (0.135)	0.175 (0.114)	28	1.664	0.107
L5-S1	0.163 (0.087)	0.161 (0.078)	28	0.035	0.972

## Discussion

The findings of this descriptive cross-sectional study suggest that there is separation of the spinous processes at L3-L4, L4-L5, and L5-S1 during CFD treatment on healthy participants, without LBP, and that this separation could be observed and measured with ultrasound. Statistical analysis revealed a significant difference in separation distance between the treatment phase to pre-treatment phase. This is an important finding demonstrating the separation of spinous process during the treatment. Gender differences were not significant, while age and BMI showed moderate correlation with separation changes. Validation in patients with low back pain is needed to assess clinical relevance.

Kinematic assessment of the spine is an important component of the overall understanding of biomechanical contributors to and effects of back pain [47]. Musculoskeletal specialists, including chiropractors, rely on changes of the normal motion patterns of the spine, among other findings, to develop a working diagnosis and a proposed treatment approach which follows evidence-based guidelines [26, 48]. Range of motion testing, orthopedic examination, palpation, and radiographic analysis are all elements of a routine spinal examination [49].

Advanced imaging techniques are useful for making quantitative and reliable measurements. However, they come with significant drawbacks [50]. Video fluoroscopy and radiography emit ionizing radiation, which is harmful to living organisms [8, 50]. These limitations necessitate the development of a technique that is safe, simple to use, relatively inexpensive, and practical in a clinical setting to measure the segmental range of motion of the spine to understand aberrant spinal dynamics [50]. Ultrasound employs non-ionizing sound waves, which are free of risk and have demonstrated good reliability in measuring spinous process distance [8–10]. Ultrasound measures the increase in the interspinous space under variable force applications of SMM, enabling chiropractic physicians to determine the force that delivers a superior clinical outcome [20]. Changes in interspinous intervals observed through in vivo ultrasound may further our understanding of spinal motion and provide insights into the clinical outcomes associated with various treatment interventions [14, 50–52].

FDT, a table-assisted, low-velocity, low-amplitude form of manual therapy, is used by over 50% of chiropractic physicians, as well as some osteopathic physicians, medical physicians, physical therapists, and other manual therapists [34–37, 53]. Many practitioners who use FDT to treat lumbar spine-related issues rely on *feel* or intuition to perform the mechanics of flexion distraction, guided by personal bias and experience. CFD is the only known form of FDT to employ well-defined treatment protocols, specifying the number and velocity of repetitions and sets based on the presence of symptoms [26].

Given that the findings of this study suggest that measurable movement of the spinous process can be achieved with ultrasound guided imaging, it would be prudent to investigate spinous process movement during the application of CFD in patients with spine pain in order to correlate optimal forces with clinical outcomes. Considering the demonstrated benefits of CFD, future investigation using CFD with ultrasound imaging could also be beneficial in understanding treatment approaches for individuals with spine pain.

### Limitations

The authors acknowledge several limitations that should be considered. The study included a small sample size of participants who were generally healthy and did not have LBP. In addition, the applied forces were not quantified in the study. Furthermore, the involvement of two different clinicians could have led to variations in the forces applied by each clinician. Additionally, one ultrasonographer performed one measurement per segment for each participant, which did not allow for generalizability. Follow-up studies should be carried out with larger sample sizes that also include patients with LBP and should quantify the applied forces and correlate them with clinical outcomes.

### Conclusions

This study used ultrasound to measure the distance between spinous processes before, during, and after the chiropractic procedure, CFD, Protocol 1. The findings of this descriptive cross-sectional study suggest that the spinous separation at L3-L4, L4-L5, and L5-S1 could be observed and quantified with ultrasound during CFD, Protocol 1, on healthy participants with no LBP. Statistical analysis showed a significant increase in separation distance from pre-to treatment phase, highlighting spinous separation during the intervention. Gender differences were not significant, while age and BMI showed moderate correlations. Future studies should include patients with LBP to observe this phenomenon and determine whether the spinous process separation correlates with clinical outcomes.

## Data Availability

The datasets used and/or analyzed during the current study are available from the corresponding author on reasonable request.

## Abbreviations

CFD: Cox^®^ flexion distraction
SD: standard deviation
L3-S1: Lumbo-sacral spine
LBP: Low back pain
MRI: Magnetic resonance imaging
SMM: Spinal manipulation and mobilization
SD: Standard deviation
